# Optic Neuritis After a Snakebite: A Diagnostic Dilemma

**DOI:** 10.31486/toj.19.0014

**Published:** 2021

**Authors:** Jeyhan Dhabhar, Varshil Mehta, Nimit Desai

**Affiliations:** ^1^ Department of Internal Medicine, MGM Medical College, Navi Mumbai, India; ^2^ Department of Cardiology, Lister Hospital, Stevenage, UK; ^3^ Department of Neurology, Medical University of South Carolina, Charleston, SC, USA

**Keywords:** *Delayed diagnosis*, *elapidae*, *neurotoxicity syndromes*, *snake bites*

## Abstract

**Background:** Snakebite is one of the major causes of morbidity and mortality in India, particularly in rural regions. Of the 57 known venomous species of snakes in India, the 4 most dangerous snakes are the cobra, the common krait, the Russell viper, and the saw-scaled viper. Of these, the snakes commonly implicated with neurotoxicity are the cobra and the common krait—both elapidae. Acute neuromuscular weakness with respiratory system involvement is the most lethal neurotoxic effect.

**Case Report:** A 24-year-old female was brought to the emergency department in an unresponsive state with a history of snakebite on the left foot. The patient was intubated, mechanically ventilated, and promptly started on snake antivenom and anticholinesterase agents. The patient improved significantly and was extubated. On day 6, she developed blurred vision and slurred speech. She was diagnosed with bilateral optic neuropathy and treated with repeat snake antivenom and steroids. She improved significantly and was discharged on day 14.

**Conclusion:** When a person is bitten by a venomous snake, antivenom is the mainstay of treatment, but clinicians must also consider possible reactions and complications. Optic neuritis following a snakebite is rare but does occur. The prognosis is generally good if clinical suspicion for such a complication is strong, the snake is identified, and the patient receives timely treatment with steroids.

## INTRODUCTION

In India, a developing country where the principal occupation is agriculture, snakes and other reptiles form an important part of the ecosystem. The World Health Organization (WHO) has classified snakebite as a neglected tropical disease of global importance.^1^ Kasturiratne et al estimated that at least 1.2 million snakebites occur annually worldwide, with approximately 421,000 envenomings and 20,000 deaths.^2^ In India, the estimated number of snakebites is higher than reported figures, as many victims choose traditional therapies and most die outside of hospitals.^3^

India has more than 242 snake species, 57 of which are highly poisonous and venomous. The 4 most dangerous species are the Indian cobra (*Naja naja*), the common krait (*Bungarus caeruleus)*, the Russell viper (*Daboia russelii)*, and the saw-scaled viper (*Echis carinatus)*.^4^

A well-known result of envenoming by kraits (*Bungarus* spp) and cobras (*Naja* spp) is neurotoxicity. Acute neuromuscular paralysis is the primary presentation of neurotoxicity and is a major cause of morbidity and mortality related to snakebites. However, timely administration of snake antivenom and ventilatory assistance can help prevent mortality and morbidity.^4^ We describe the case of a 24-year-old female who presented with snakebite from an Indian cobra.

## CASE REPORT

A 24-year-old female was brought to the emergency room in an unresponsive and gasping state. She had been bitten by a snake on the dorsal aspect of her left foot approximately 1 hour before presentation. The patient sustained the snakebite when she was walking in a garden. Approximately 15 to 20 minutes following the bite, she developed bilateral severe ptosis followed by dyspnea that was rapidly progressive, explaining why the patient was gasping for breath when she reached the hospital. She had no significant medical history or comorbidities. The patient's relatives brought the snake to the hospital, and it was identified as an Indian spectacled cobra (Figure, left panel).

**Figure.**
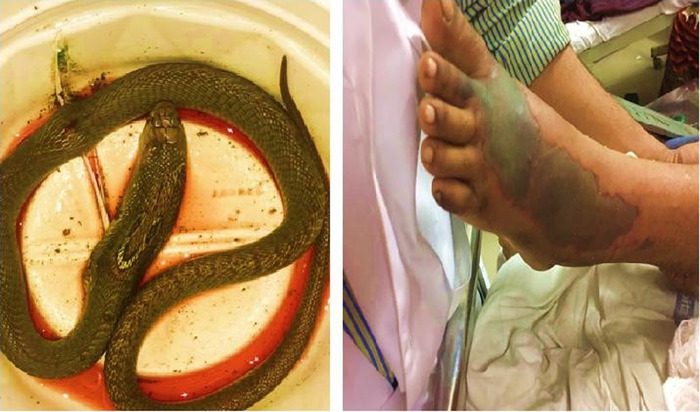
**(Left) The Indian spectacled cobra that bit the patient. (Right) Cellulitis developed at the bite site by day 7.**

The patient's oxygen saturation (SpO_2_) was 70% to 80% on high-flow oxygen. Other vitals were stable. Physical examination was unremarkable except for the bite marks on the left foot. Peripheral pulses were felt in all 4 limbs. Because of the patient's respiratory distress, she was intubated and provided with ventilatory support.

Whole blood clotting test was normal. The patient was started on polyvalent snake antivenom (snake venom antiserum IP [Indian pharmacopeia], lyophilized) 10 vials diluted in 500 mL of 5% dextrose, administered 3 doses of intravenous (IV) neostigmine + atropine, and transferred to the intensive care unit (ICU).

On day 2, a repeat dose of snake antivenom was administered, and the patient showed signs of improvement. On day 3, the patient was conscious and oriented, and she was extubated. Postextubation, the patient maintained SpO_2_ of 96% on ambient air. Because of the progressive improvement in the patient's condition, she was transferred to the wards on day 4. However, at the site of the bite, a reddish hue progressively developed, in addition to swelling and an increase in local temperature. On day 6, the patient suddenly developed severely blurred and diminished vision associated with ptosis and slurred speech. Full neurologic examination found no neurologic deficit other than the visual findings: the patient had bilateral ptosis and only perceived light in both eyes. Computed tomography scan and magnetic resonance imaging were normal. After consultation with the ophthalmologist and neurologist, delayed neurotoxicity from the venom was suspected, and an additional dose of snake antivenom was administered. Visual evoked potential indicated bilateral axonopathic optic neuropathy with maculopathy indicative of optic neuritis. The patient was started on IV methylprednisolone 500 mg daily for 7 days and then transitioned to oral prednisone 40 mg 3 times daily that was tapered by 20 mg every 7 days. By day 7, the swelling had become tender, indicative of cellulitis (Figure, right panel). IV antibiotics amoxicillin clavulanate 1.2 g twice daily and metronidazole 500 mg 3 times daily were administered, the limb was elevated, and a magnesium sulphate dressing was applied.

The patient showed gradual improvement in her symptoms by day 4 of IV methylprednisolone and was discharged 14 days after initial presentation. After discharge, the patient was followed monthly for 3 months. She fully recovered her visual acuity within 1 month. She showed no residual effects and resumed her job and daily activities.

## DISCUSSION

Our patient initially appeared to be a straightforward, classic case of neurotoxic snakebite without an accompanying diagnostic dilemma. The patient was treated per WHO guidelines^5^ and rapidly recovered from her initial symptoms. What was baffling was her deterioration on day 6 and the development of optic neuropathy. We found only 7 case reports of optic neuritis following snakebite.^6-12^ All 7 patients had received snake antivenom. As with our patient, the deterioration of vision occurred on day 6 in 3 of the reported cases.^7-9^

Hypotheses for the cause of optic neuritis after snakebite are the snake venom itself,^11^ extensive hemorrhages, allergy to snake antivenom,^9^ and capillary damage.^8^ Indirect evidence supports each theory. For example, Mathur published a case of optic neuritis following the administration of snake antivenom after a nonpoisonous snakebite and claimed that the antivenom led to the optic neuritis.^9^ Rao reported the sudden development of blindness following a cobra bite and attributed it to the toxic effects of the venom.^11^

In our case, we were unable to ascertain if a reaction to the snake antivenom or the direct toxic effect of the snake venom was the cause of the patient's optic neuritis. Irrespective of the etiology of optic neuritis, the treatment is the same: steroids. Steroids were used to treat optic neuritis following snakebite in 5 of the 7 reported cases. The patients’ vision was reported to improve in all cases, but improvement took longer for the 2 patients who did not receive steroids.

Neuropathy is a known delayed complication of envenomation.^13^ Neuropathy is often first noticed after recovery from the acute phase of ventilation and ICU care. In a study of 210 patients, 38 patients who were bitten by the common krait developed delayed neurologic deficits.^13^ Subsequently, 14 of them also developed nerve conduction defects that persisted from 2 weeks to 6 months, followed by complete recovery. After the acute phase of envenoming, polyneuropathy has been observed for several months in snake-bitten patients.^14^ Cases of Guillain-Barré syndrome have also been reported.^15^

Further study and evaluation are needed to identify the factors responsible for long-term neurologic effects. Possible explanations include delayed immune-mediated reactions to toxins or antivenom and ongoing axonal damage caused by neurotoxins.

## CONCLUSION

Snake antivenom is the mainstay of treatment for venomous snakebite, but clinicians should keep in mind the possible reactions and complications, both immediate and delayed. The treating physician should not become complacent once the patient starts to improve but should be alert to delayed manifestations. Optic neuritis following snakebite is rare but does occur. The prognosis is generally good, particularly if steroids are administered.

## Notes

This article meets the Accreditation Council for Graduate Medical Education and the American Board of Medical Specialties Maintenance of Certification competencies for Patient Care and Medical Knowledge.
